# Pediatric Upper-Lip Avulsion Reconstructed With an Abbé Flap Following a Dog Bite

**DOI:** 10.7759/cureus.108004

**Published:** 2026-04-29

**Authors:** Guru Bhushan, Priyank Rai, Lokesh Chandila, Lina Priya, Sourabh Jain

**Affiliations:** 1 Department of Plastic and Reconstructive Surgery, Pacific Medical College and Hospital, Udaipur, IND; 2 Department of Oral and Maxillofacial Surgery, Pacific Dental College and Research Center, Udaipur, IND

**Keywords:** dog bite wounds, facial tissue loss, graft, twisted pedicle, young child

## Abstract

Dog-bite injuries are a common cause of pediatric facial trauma and may result in complex soft-tissue defects. This report describes a three-year-old child with a full-thickness avulsion of the right upper lip associated with an anterior maxillary alveolar fracture following a dog bite. Surgical management included reconstruction using a lower-lip Abbé flap along with stabilization of the alveolar fracture using interdental wiring. Thorough wound debridement, irrigation, and appropriate antimicrobial and rabies prophylaxis were performed. The flap remained viable, and pedicle division was carried out on postoperative day 19. Healing was uneventful, with restoration of oral competence, normal feeding ability, satisfactory lip symmetry, and minimal vermilion mismatch. This case demonstrates that early reconstruction with an Abbé flap, combined with management of associated skeletal injury, is a feasible approach in such presentations. Further studies are required to evaluate long-term outcomes.

## Introduction

Dog-bite injuries are a significant cause of maxillofacial trauma in children and account for a substantial proportion of pediatric emergency visits worldwide. Young children are particularly vulnerable due to their short stature, limited defensive reflexes, and close proximity of the face to animals, resulting in a higher incidence of facial involvement [1]. The lips are especially susceptible because of their prominence, and injuries in this region often result in complex soft-tissue defects with important functional and esthetic consequences [2,3]. Lip avulsion injuries are an uncommon but severe subset of facial trauma, most often resulting from dog bites, with pediatric patients, especially younger children, showing a higher predilection for head and facial involvement, often requiring advanced reconstructive procedures in up to 25% of cases [4].

The management of dog-bite wounds has historically been controversial, particularly regarding the timing of closure due to concerns of infection. However, current evidence supports primary repair of facial bite wounds following thorough irrigation, adequate debridement, and appropriate antibiotic coverage, with favorable healing outcomes attributed to the rich vascularity of facial tissues [5]. Various reconstructive techniques have been described for lip defects, ranging from primary closure and local advancement flaps to more complex regional and free tissue transfers. Among these, the Abbé flap (cross-lip flap) is a well-established option for reconstructing central and lateral lip defects, providing good tissue match and preservation of orbicularis oris function [6,7]. The present case describes the successful management of a pediatric full-thickness upper-lip avulsion caused by a dog bite using a lower-lip Abbé flap, with emphasis on the surgical approach and clinical outcome.

## Case presentation

**Figure 1:**
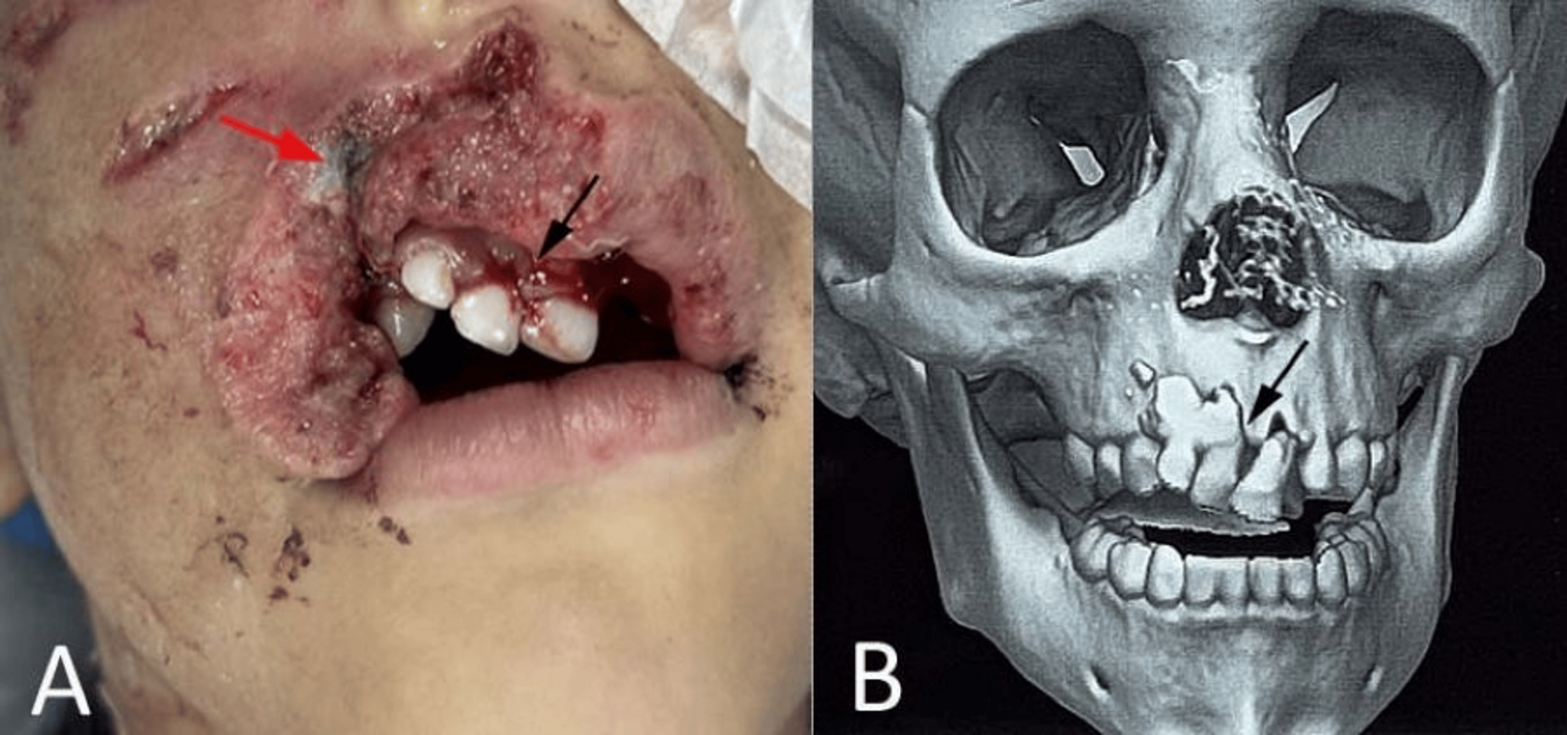
Clinical and radiologic findings of upper-lip avulsion and associated anterior maxillary alveolar fracture following a dog bite. (A) Facial soft tissue avulsion of the upper lip following a dog bite (red arrow) and (B) computed tomography (CT) scan showing anterior maxillary alveolar fracture with displacement of maxillary anterior teeth (black arrows).

A three-year-old female child presented to the emergency department with a history of facial trauma following a dog bite by a stray animal approximately 6 h prior to admission. The child was conscious, alert, and hemodynamically stable at presentation, with no history of loss of consciousness or associated systemic injury. Clinical examination revealed a full-thickness avulsion defect involving the right upper lip measuring approximately 2×1.5 cm. The defect included loss of the skin, vermilion, orbicularis oris muscle, and mucosa, extending laterally toward the right oral commissure, which appeared partially preserved but distorted (Figure 1, panel A). The wound margins were irregular and contaminated, which is consistent with the avulsive nature of the injury. Intraoral examination revealed mobility and tenderness in the right anterior maxillary alveolar segment, raising the suspicion of an underlying alveolar fracture. No dental avulsion or additional intraoral soft-tissue injuries were noted.

**Figure 2:**
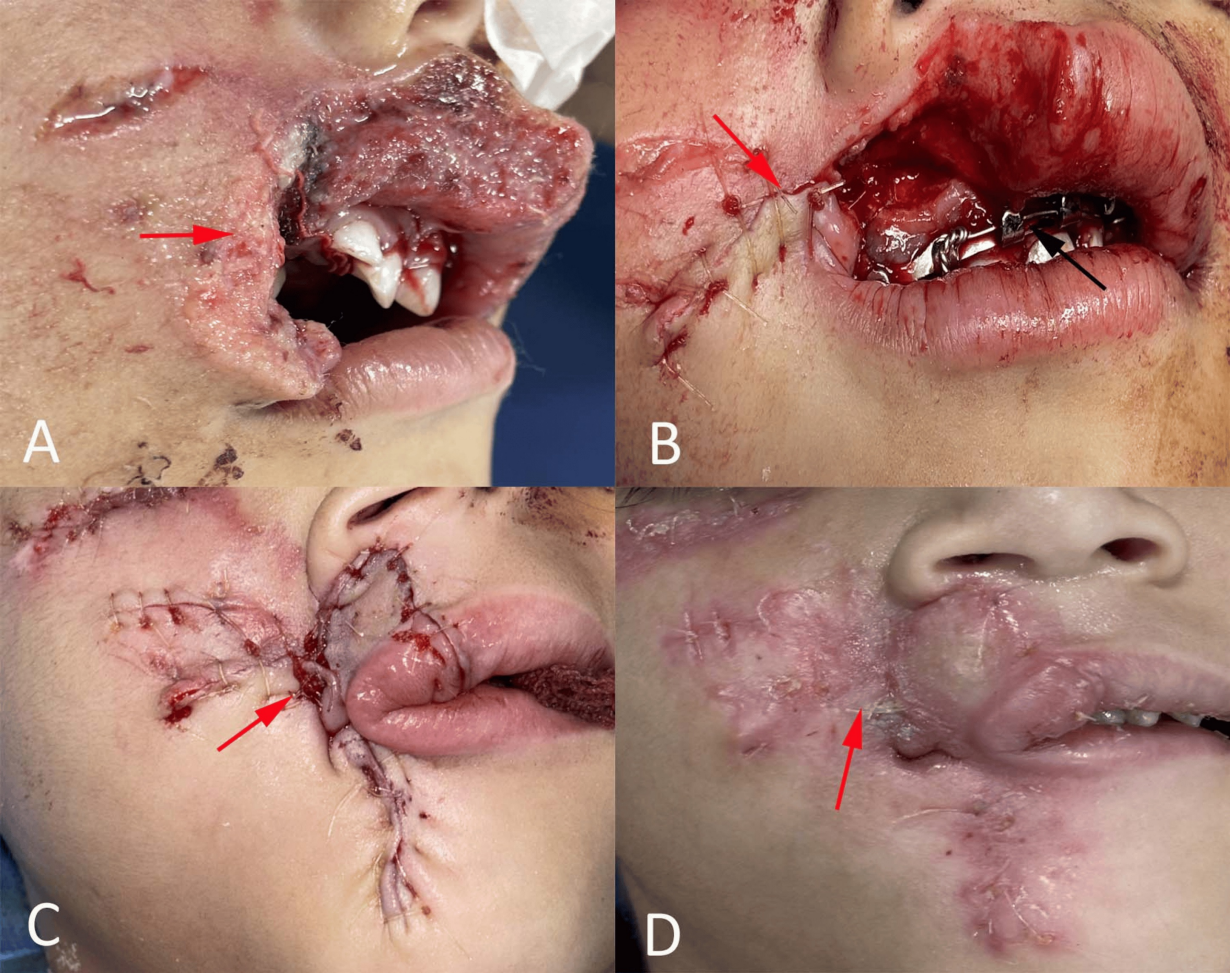
Sequential intraoperative and postoperative views demonstrating reconstruction of the upper-lip defect using an Abbé flap. (A) Intraoperative view following wound debridement, (B) stabilization of anterior maxillary alveolar fracture using interdental wiring, (C) rotation and inset of Abbé flap into the upper-lip defect (red arrows), (D) postoperative appearance showing viable flap and healed surgical site.

Radiographic evaluation using three-dimensional computed tomography (CT) confirmed disruption of the right anterior maxillary alveolus without involvement of the other facial bones (Figure 1, panel B). Based on clinical and radiographic findings, a diagnosis of full-thickness upper-lip avulsion secondary to a dog bite with an associated anterior maxillary alveolar fracture was established. Considering the extent of tissue loss and the need for functional and esthetic rehabilitation, early surgical reconstruction using a cross-lip Abbé flap was planned. The procedure was performed under general anesthesia with nasoendotracheal intubation. Local infiltration with 2% lignocaine containing adrenaline (1:200,000) was administered to achieve hemostasis. Rabies immunoglobulin was infiltrated locally around the wound margins in accordance with standard prophylactic guidelines. The wound was irrigated thoroughly with copious amounts of normal saline, and all devitalized tissues were carefully debrided while preserving viable structures (Figure 2, panel A). The anterior maxillary alveolar fracture was reduced and stabilized using interdental stainless-steel wiring to restore occlusal alignment and ensure immobilization (Figure 2, panel B).

A full-thickness Abbé flap was designed on the lower lip, based on the inferior labial artery. The flap incorporated the skin, vermilion, orbicularis oris muscle, and mucosa to provide like-for-like tissue reconstruction. After careful elevation, the flap was rotated and inserted into the upper lip. Particular attention was paid to the precise alignment of the vermilion border and white roll to achieve optimal esthetic outcomes (Figure 2, panel C). Layered closure was performed using absorbable sutures for the muscle and mucosa and fine nonabsorbable sutures for the skin. The donor site was primarily closed. The lower-lip donor site was closed in layers with precise muscle and vermilion alignment, and healing was uneventful without functional deficit or significant scarring. Postoperatively, the patient was administered intravenous antibiotics, analgesics, and tetanus prophylaxis. The rabies vaccination schedule was completed according to the protocol. Oral feeding was initiated with a liquid diet and gradually advanced to a soft diet over the course of one week.

Pedicle division was performed on postoperative day 19. The flap demonstrated good vascularity and successful integration into the recipient site (Figure 2, panel D). Healing was uneventful, with the restoration of oral competence and satisfactory functional outcomes. At follow-up, the patient exhibited adequate lip symmetry, with minimal vermilion mismatch. The oral commissure remained functionally competent, and the feeding ability was fully restored. Minor esthetic refinements may be considered in the future as children grow older. At follow-up, the patient demonstrated adequate oral competence with no drooling, normal feeding ability, and age-appropriate speech development, indicating satisfactory functional recovery.

## Discussion

Dog-bite injuries to the face constitute a significant proportion of pediatric maxillofacial trauma, with the lips commonly affected due to their prominence and exposed position [1,8]. Epidemiological studies indicate that children are particularly vulnerable, frequently presenting with complex injuries including avulsions and tissue loss [8]. Abuabara and Piccart et al. reported that the head and neck region accounts for the majority of dog-bite injuries, with frequent involvement of the perioral region, emphasizing the reconstructive challenges associated with these cases [9,10].

Full-thickness lip avulsion injuries are particularly complex as they involve disruption of multiple anatomical layers and compromise oral competence and facial expression [4,7]. In pediatric patients, additional considerations such as facial growth, scar maturation, and long-term oral competence further complicate treatment planning [4]. In the present case, the defect extended toward the oral commissure and was associated with an anterior maxillary alveolar fracture, increasing the complexity of surgical management.

Lip avulsion injuries can be classified based on extent (partial vs. complete), thickness (partial- vs. full-thickness), and anatomical involvement (central, lateral, or commissural). Full-thickness avulsions involve loss of skin, vermilion, orbicularis oris muscle, and mucosa, resulting in significant impairment of oral competence, speech, mastication, and facial expression. The complexity increases when the defect extends toward the commissure or is associated with underlying skeletal injuries. Management depends on defect size and location, ranging from primary closure and local advancement flaps for small defects to regional flaps such as the Abbé or Estlander flap for larger defects, aimed at restoring both function and esthetics. In pediatric patients, additional considerations include facial growth, scar maturation, and long-term functional outcomes, making early, well-planned reconstruction essential [4,6,7,9].

Historically, bite wounds were often managed with delayed closure due to concerns regarding infection. However, more recent evidence supports immediate repair following meticulous irrigation, debridement, and appropriate antibiotic coverage [1,11,12]. Chen et al. demonstrated that timely intervention in facial bite injuries is associated with favorable healing and low infection rates, largely attributed to the rich vascularity of facial tissues [11]. Similar observations have been reported by Cavalcanti et al. and Sikka et al., who highlighted improved outcomes in pediatric facial trauma managed with prompt surgical care. The present case aligns with these findings, as no postoperative infection or wound-related complications were observed [1,12].

Among the various reconstructive options available for lip defects, the Abbé flap remains a well-established technique for central and lateral lip reconstruction [6,7,13]. It provides tissue that closely matches the native lip in color, texture, and thickness, while preserving the continuity of the orbicularis oris muscle. Hahn et al. emphasized the role of cross-lip flaps in restoring lip symmetry and dynamic function [7], while Erol et al. highlighted their versatility in managing complex deformities [13]. In this case, the use of a lower-lip Abbé flap enabled precise reconstruction of the defect with satisfactory integration at the recipient site.

A notable aspect of this case was the simultaneous management of the associated anterior maxillary alveolar fracture. Stabilization using interdental wiring helped restore occlusal alignment and provided a stable foundation for soft-tissue repair. Such combined management has been infrequently reported in the literature, highlighting the importance of addressing both skeletal and soft-tissue components in a coordinated manner [1,11].

The postoperative course was uneventful, with adequate healing and preservation of lip symmetry and oral competence. Delayed pedicle division on postoperative day 19 did not compromise flap viability, indicating a reliable vascular supply. Froind et al. have also emphasized the importance of comprehensive evaluation in pediatric dog-bite injuries to rule out associated complications, further supporting the need for thorough clinical and radiographic assessment [14]. While the Abbé flap is a well-established technique for lip reconstruction, the uniqueness of the present case lies in the simultaneous management of a pediatric full-thickness upper-lip avulsion and an associated anterior maxillary alveolar fracture in a single-stage procedure. Most reported cases in the literature address soft-tissue reconstruction and bony injuries separately or in staged approaches. In contrast, the current approach demonstrates the feasibility and effectiveness of early, combined reconstruction, enabling restoration of both soft-tissue form and occlusal stability in a single operative setting. This integrated management is particularly significant in pediatric patients, where minimizing surgical interventions and exposure to anesthesia is critical while also supporting optimal functional recovery and facial growth.

Despite the favorable outcome, this report represents a single clinical case with short-term follow-up. The follow-up period in the present case was limited, which precludes a comprehensive assessment of long-term outcomes such as facial growth, scar maturation, speech development, and functional stability. However, early postoperative results demonstrated satisfactory healing and restoration of oral competence. Long-term follow-up is planned to evaluate growth-related changes and functional outcomes over time. Further studies involving larger cohorts are required to establish standardized protocols for managing similar injuries.

## Conclusions

This case highlights the successful reconstruction of a pediatric upper-lip avulsion using an Abbé flap with simultaneous stabilization of an associated alveolar fracture. In this patient, early primary reconstruction appeared feasible and resulted in satisfactory functional and esthetic outcomes. While this approach may be considered in similar clinical scenarios, further studies with larger samples and long-term follow-up are required to establish its generalizability.
